# Endurance Is Improved in Female Rats After Living High-Training High Despite Alterations in Skeletal Muscle

**DOI:** 10.3389/fspor.2021.663857

**Published:** 2021-05-28

**Authors:** Alexandra Malgoyre, Alexandre Prola, Adelie Meunier, Rachel Chapot, Bernard Serrurier, Nathalie Koulmann, Xavier Bigard, Hervé Sanchez

**Affiliations:** ^1^Département des Environnements Opérationnels, Institut de Recherche Biomédicale des Armées, Brétigny-sur-Orge, France; ^2^Laboratoire de Biologie de l'Exercice pour la Performance et la Santé, Université Evry, Université Paris Saclay, Evry, France; ^3^Department of Cell Physiology and Metabolism, Faculty of Medicine, University of Geneva, Geneva, Switzerland; ^4^Ecole du Val de Grâce, Paris, France

**Keywords:** mitochondrial respiration, oxidative capacity, fatty acid, skeletal muscle, chronic hypoxia, endurance training, rat

## Abstract

Altitude camps are used during the preparation of endurance athletes to improve performance based on the stimulation of erythropoiesis by living at high altitude. In addition to such whole-body adaptations, studies have suggested that high-altitude training increases mitochondrial mass, but this has been challenged by later studies. Here, we hypothesized that living and training at high altitude (LHTH) improves mitochondrial efficiency and/or substrate utilization. Female rats were exposed and trained in hypoxia (simulated 3,200 m) for 5 weeks (LHTH) and compared to sedentary rats living in hypoxia (LH) or normoxia (LL) or those that trained in normoxia (LLTL). Maximal aerobic velocity (MAV) improved with training, independently of hypoxia, whereas the time to exhaustion, performed at 65% of MAV, increased both with training (*P* = 0.009) and hypoxia (*P* = 0.015), with an additive effect of the two conditions. The distance run was 7.98 ± 0.57 km in LHTH vs. 6.94 ± 0.51 in LLTL (+15%, ns). The hematocrit increased >20% with hypoxia (*P* < 0.001). The increases in mitochondrial mass and maximal oxidative capacity with endurance training were blunted by combination with hypoxia (−30% for citrate synthase, *P* < 0.01, and −23% for Vmax _glut−succ_, *P* < 0.001 between LHTH and LLTL). A similar reduction between the LHTH and LLTL groups was found for maximal respiration with pyruvate (−29%, *P* < 0.001), for acceptor-control ratio (−36%, hypoxia effect, *P* < 0.001), and for creatine kinase efficiency (−48%, *P* < 0.01). 3-hydroxyl acyl coenzyme A dehydrogenase was not altered by hypoxia, whereas maximal respiration with Palmitoyl-CoA specifically decreased. Overall, our results show that mitochondrial adaptations are not involved in the improvement of submaximal aerobic performance after LHTH, suggesting that the benefits of altitude camps in females relies essentially on other factors, such as the transitory elevation of hematocrit, and should be planned a few weeks before competition and not several months.

## Introduction

Altitude training has been used for decades by endurance athletes to improve sea-level performance. Various modalities of combining hypoxia exposure and training have been proposed (Millet et al., 2010; Girard et al., 2020).

Living at high altitude (LH), regardless of the conditions of training, is an established method to improve aerobic performance, mainly due to erythrocytosis (Hahn and Gore, 2001; Stray-Gundersen and Levine, 2008; Gore et al., 2013) but other mechanisms, such as improvements in running economy, glycolysis, and buffering capacity, have also been suggested (Gore et al., 2007). Training at low altitude (TL) has long been preferred to maintain exercise intensity, which is not possible at high altitude due to reduced oxygen flow and could be responsible for relative detraining (Levine and Stray-Gundersen, 1997; Levine, 2002). Such a strategy of “living high-training low” (LHTL) would benefit whole-body adaptations related to altitude acclimation while maintaining the same intensity and training load.

More recently, the effects of training in hypoxia (TH) on skeletal muscle tissue have been studied (Hoppeler et al., 2008; Lundby et al., 2009). Local muscle hypoxia was suggested to be an important stimulus during exercise (Richardson et al., 1999) and it has been argued that it may be a way to increase alterations of cellular homeostasis induced by exercising and, thus, a relevant manner to potentiate greater muscular adaptations than by TL (Hawley et al., 2018). Thus, a more pronounced transcriptional response of genes in skeletal muscle was already shown with TH and suggested that they are oxygen sensitive (Vogt et al., 2001; Zoll et al., 2006). These genes are, at least partially, regulated by hypoxia-inducible factor-1α (HIF-1α), the main transcription factor involved in cellular responses induced by hypoxia. Thus, angiogenesis mediated through vascular endothelial growth factor (VEGF) has been hypothesized for the efficiency of “living low-training high” (LLTH), but several studies have also suggested improved oxidative capacity: (i) elevation of mitochondrial density (Desplanches et al., 1993; Vogt et al., 2001; Schmutz et al., 2010; Jacobs and Lundby, 2013), (ii) enhanced oxidative and metabolic gene expression (Vogt et al., 2001; Zoll et al., 2006; Schmutz et al., 2010), and (iii) increased citrate synthase (CS) activity (Melissa et al., 1997) after TH. Nevertheless, this issue is still debated (Vogt and Hoppeler, 2010) and several studies found absolutely no benefit on mitochondrial mass (Bakkman et al., 2007; Desplanches et al., 2014; Robach et al., 2014). Moreover, animal models have suggested that HIF-1α may even suppress oxidative metabolism and limit the response to training (Mason et al., 2007; Lindholm and Rundqvist, 2016). More subtle and qualitative adaptations should also be considered, such as mitochondrial coupling and efficiency (Howald et al., 1990; Ponsot et al., 2006; Desplanches et al., 2014) or substrate utilization between lipids and carbohydrates (Roels et al., 2007; Robach et al., 2014).

The effects of chronic hypoxia on skeletal muscle, are also debated. The prolonged severe hypoxia typical of expeditions to the Himalayas leads to decreased mitochondrial density and/or impediment of mitochondrial function (Green et al., 1989; Howald et al., 1990; MacDougall et al., 1991; Jacobs et al., 2012b; Levett et al., 2012) but the effects of a lower level and shorter duration of hypoxia are not clear. Recent studies do not support alterations of mitochondrial function and content after only a few weeks of passive exposure to moderate hypoxia (Jacobs et al., 2012a). Thus, Murray (2016) expressed the importance of considering the duration and extent of hypoxia exposure.

Combining the benefit of certain skeletal muscle adaptations observed with LLTH and the increase of hemoglobin mass (Hbmass) obtained with LH could be beneficial for endurance, although this is still debated (Robach et al., 2012; Millet et al., 2019) and provided that the impediment of mitochondrial function described under conditions of severe hypoxia is prevented. In addition, LHTH is the most pragmatic situation for athletes during altitude training camps. However, very few studies conducted under LHTH conditions have focused on mitochondrial adaptations in muscle (Bigard et al., 1991; Galbes et al., 2008) and with a single one in humans (Desplanches et al., 1996). Nevertheless, a recent comprehensive review suggested (Horscroft and Murray, 2014) that fatty-acid oxidation could be specifically impeded, even in moderate hypoxia, as previously described in humans (Roberts et al., 1996) and mice (Morash et al., 2013), whereas maximal lipid oxidation is a decisive factor for endurance. However, other animal studies found that short-duration intermittent hypoxia associated with endurance training enhanced fatty-acid metabolism in skeletal muscle (Suzuki, 2016) and we showed an increased affinity for fatty acids in glycolytic and oxidative-slow twitch muscles (Malgoyre et al., 2017), which could provide an advantage for low-intensity exercise.

We aimed to know whether mitochondrial alterations in muscle induced by endurance training are enhanced or limited by a moderate hypoxia in a model of LHTH with female rats showing no body weight alteration in this condition. The main purpose of the present study was to examine the effects of hypoxic endurance training on aerobic performance and quantitative and qualitative mitochondrial changes in the *plantaris* muscle, a glyco-oxidative muscle that is highly recruited during running.

## Materials and Methods

### Animals and Experimental Design

This study was performed in accordance with both the Helsinki Declaration concerning the treatment of laboratory animals and the European Convention for the Protection of Vertebrate Animals used for Experimental and other Scientific Purposes (Council of Europe no. 129, Strasbourg, 1985). It was approved by our local animal ethics committee. We used female Wistar rats (weighing 170–200 g) obtained from Charles River Laboratories (L'Arbresle, France). All animals were housed by two per cage and subjected to an artificial 12-h light/12-h dark cycle. After 1 week of housing, the rats were randomly assigned to one of four experimental groups (*n* = 8 each): either sedentary or trained in either a hypoxic or normoxic environment. Thus, four situations were represented: LL for sedentary and living low, LH for sedentary and living high, LLTL for trained and living low, LHTH for trained and living high. Animals from the hypoxic groups were housed in a hypobaric chamber (T.I.M., Marseille, France) in which the barometric pressure was progressively reduced to 500 mmHg/666 hPa, values, nearly equivalent to an elevation of 3,200 m. They were maintained under hypobaric conditions for 5 weeks, at an ambient temperature of 22 ± 2°C. Both normoxic and hypoxic rats had free access to water and standard laboratory chow in powder form (AO3 UAR, Charles River, Les Oncins, France). The pressure of the hypobaric chamber was elevated to that of sea level once a day, at which time the rats were weighed. The LHTH group was trained under normobaric hypoxia in a tent in which the oxygen level was decreased to a FiO_2_ of 14% through a CAT 12 air-unit from Colorado Altitude Training® (Louisville, USA). The PiO_2_ was maintained at 100 mmHg/133 hPa under these two simulated altitude conditions, whether normobaric or hypobaric.

### Tissue and Blood Processing

At the end of the 5-week period and 48-h after the last exercise session, animals were anesthetized with an intraperitoneal injection of pentobarbital sodium (50 mg/100 g body weight). Blood samples were withdrawn from the abdominal aorta with a heparinized syringe and a portion analyzed for hematocrit. The *plantaris* muscles were excised immediately before exsanguination. The muscles from the right side of the body were immersed in Krebs solution (118 mM NaCl, 25 mM NaHCO_3_, 4.7 mM KH_2_PO_4_, and 1.2 mM MgSO_4_) for mitochondrial respiration experiments and those from the left side of the body were rapidly frozen in liquid nitrogen for biochemical and RT-PCR assays.

The abdominal and retroperitoneal fat masses were measured by meticulous manual skinning of white adipose tissue. The entire fat mass surrounding the kidneys was removed and the adrenal glands withdrawn before weighing the fat mass on a high-precision balance. The heart was removed and the left and right ventricular isolated after atrial and septum resection before weighing.

### Training Sessions

Training consisted of running sessions on a treadmill 5 days a week for 5 weeks. The training intensity was progressively increased and adapted to the environment in a way that the relative intensity was similar under normoxia and hypoxia.

Each session under normoxia corresponded to running at a speed of 25.5 m.min^−1^ with a 10-degree slope for 45–60 min the first 2 weeks. Previous data from our laboratory or others (Gonzalez et al., 1993; Wehrlin and Hallen, 2006) estimated that the maximal aerobic capacity was reduced by ~6.3% per 1,000 m, hence, almost 20% at 3,200 m or 14% FiO_2._ Thus, the speed was reduced to 20.4 m.min^−1^ for hypoxic training. The intensity for the last 3 weeks was increased to 30 m.min^−1^ under normoxia and 24.6 m.min^−1^ under hypoxia and the duration extended from 60 to 80 min.

### Assessment of Aerobic Performance

Aerobic performance was assessed by measuring both maximal aerobic and endurance capacity. The performance of each rat was analyzed by measuring their maximal aerobic velocity (MAV), in which the speed was progressively increased. After a 6-min warm-up at 13.6 m.min^−1^, the speed of the treadmill was increased by 3.5 m.min^−1^ each 2 min up to 34.5 m.min^−1^ and then by 1.7 m.min^−1^ each 90 s. The test was stopped when the rats were unable keep up with the treadmill. The speed of the last completed level was considered to be the MAV.

For each animal, the running time to exhaustion was assessed 48 h after determination of the MAV at a speed corresponding to 65% of their own MAV. Animals ran until exhaustion, determined when the animals remained near an electric grid, despite receiving three shocks. At the end of the test, blood was collected through a small incision at the tip of the tail into a vial containing EDTA and maintained on ice for subsequent biochemical tests on the plasma.

### *In situ* Study of Mitochondrial Respiration

Mitochondrial respiration was studied *in situ* in saponin-permeabilized fibers, as previously described (Kuznetsov et al., 2008). Briefly, fibers were separated under a binocular microscope in solution S (see below) at 4°C. They were then permeabilized by incubation with 50 μg.ml^−1^ saponin in solution S for 30 min and rinsed three times for 5 min in fresh solution R, containing no energy source (see below), immediately before respiratory measurements. This step ensured that adenine nucleotides, creatine phosphate (PCr), and other remaining endogenous substrates were completely washed out. For each muscle, we analyzed 3–8 mg of freshly permeabilized fibers in triplicate at 22°C with a Clark electrode (Hansatech Oxygraph Instruments, Norfolk, England) in 1.5 ml respiration solution (solution R), with continual stirring. After measurement, the fibers were removed, dried, and weighed. Respiration rates are expressed as micromoles of oxygen per minute per gram of dry weight of the fibers (μmole O_2_.min^−1^.g^−1^ dw).

Solutions R and S contained 2.77 mM CaK_2_EGTA, 7.23 mM K_2_EGTA (100 nM free Ca^2+^), 6.56 mM MgCl_2_ (1 mM free Mg^2+^), 20 mM taurine, 0.5 mM DTT, 50 mM K-methane sulfonate (160 mM ionic strength), and 20 mM imidazole (pH 7.1). Solution S also contained 5.7 mM Na_2_ATP, and 15 mM creatine-phosphate, whereas solution R also contained 3 mM K_2_HPO_4_, 10 mM Na methane sulfonate, and 6 mg.ml^−1^ fatty acid-free bovine serum albumin (BSA). This concentration of BSA was chosen based on prior optimization, following recommendations to maintain the [Palmitoyl-CoA + carnitine] to [albumin] ratio below 5, as in the classical method for the assessment of Carnitine Palmitoyl Transferase-1 (CPT-1) activity (McGarry et al., 1977; Bentebibel et al., 2006). In an extensive pilot study, we tested a range of BSA concentrations [2 mg/ml (30 μM), 4 mg/ml (60 μM), and 6 mg/ml (90 μM)] in R solution and chose 6 mg/ml BSA (ratio FA/albumin 4.4 <5), as it was required for optimal respiration in Palmitoyl-CoA.

We used two protocols to study mitochondrial metabolism. The first was a protocol with three aims: (1) determination of the rate of the maximal oxidative capacity (Vmax), (2) determination of the specific oxidation for pyruvate (V_maxPyr_), consisting of the successive addition of 2 mM pyruvate, 0.1 mM ADP, 20 mM creatine, 2 mM ADP, 10 mM glutamate, and 12 mM succinate in the presence of 4 mM malate and (3) estimation of the Km for ADP, with or without creatine, using the Michaelis Menten equation (Kuznetsov and Saks, 1986; Kuznetsov et al., 1996; Anflous et al., 2001; Ponsot et al., 2006; Perry et al., 2012):

V_*ADP*_ = (Vmax x [ADP]) / (Km + [ADP]) avec [ADP] in nM

That is Km = ((Vmax – V_*ADP*_) × [ADP])/V_*ADP*_

Calculated for Vmax = V_*ADP*_ at 2 mM and [ADP] = 100 nM with and without creatine for calculation of Km ADP with and without creatine

The second protocol, was used to measure the maximal rates of respiration with Palmitoyl-CoA, a long-chain fatty acid (Ponsot et al., 2005; Tardo-Dino et al., 2019) which must be transported across both mitochondrial membranes (via CPT-1 and CPT-2) in the presence of carnitine. We added Palmitoyl-CoA (final concentration of 400 μM) in the presence of 1 mM carnitine, 2 mM ADP, and 0.5 mM malate.

At the end of each protocol, we assessed the integrity of the mitochondrial membrane in random samples by adding cytochrome *c* as an internal control for the quality of the mitochondrial preparations. The data were excluded from the analyses if respiration increased by more than 10% relative to the previous step preceding the addition of cytochrome *c*.

### Biochemical Measurements

The non-esterified free fatty acids (FFA) glycemia and lactate were determined in plasma by enzymatic methods using an automated biomedical analyzer (Roche-Hitachi 912, Meylan, France; *n* = 8 per group).

### Enzymatic Activities

Frozen tissue was weighed to obtain ~10-mg samples. For CS, the extraction was performed in ice-cold buffer (50 mg/ml) containing 5 mM HEPES (pH 8.7), 1 mM EGTA, 1 mM DTT, 5 mM MgCl_2_, and 0.1% Triton X-100 and incubated for 60 min at 0°C to ensure complete enzyme extraction (*n* = 8 per group). CS activity was assayed at 30°C (pH 7.5) following the apparition of the mercaptide anion by spectrophotometry after the addition of oxaloacetate (50 mM) (O-4126, Sigma, France), as previously described (Srere, 1969). The extraction of 3-hydroxyl acyl coenzyme A dehydrogenase (3-HAD) was performed in ice-cold 300 mM phosphate buffer (50 mg/ml) containing KH_2_PO_4_ (pH 7.7) and 0.05% BSA. Enzyme activity was determined at 25°C by the disappearance of NADH by spectrophotometry after the addition of aceto-acetyl CoA (A-1625, Sigma, France), as previously described by Lowry and Passonneau (1972). Enzyme activities are expressed as the appearance or disappearance of substrate in micromoles per minute per wet weight (i.e., IU per gram wet weight).

### Protein Isolation and Immunoblot Analysis

Initial muscle samples frozen at −80°C were homogenized at 4°C in 15 vol buffer [50 mM Tris-HCl (pH 7.4), 100 mM NaCl, 2 mM EDTA, 2 mM EGTA, 50 mM sodium fluoride, 120 nM okadaic acid, 3 mM benzamidine, 1 mM phenylmethylsulphonyl fluoride, 1 mM DTT, 50 mM glycerophosphate, 10 μL.mL^−1^ activated sodium orthovanadate, 3 μL.mL^−1^ protease inhibitor cocktail (set III, EDTA-free, Calbiochem, Darmstadt, Germany), and 3 μL.mL^−1^ phosphatase inhibitor cocktail (set II, Calbiochem, Darmstadt, Germany)]. Homogenates were centrifuged at 12,000 × g for 20 min at 4°C. The protein concentration was determined by the bicinchoninic acid method (Roche/Hitachi 912 Instrument; Roche Diagnostics, Mannheim, Germany). Total protein (50 μg) was subjected to SDS-PAGE and transferred onto nitrocellulose membranes (Hybond C-extra, Amersham Pharmacia Biotech, Orsay, France). Equal protein loading of the lanes was confirmed by Ponceau Red staining. Membranes were incubated overnight at 4°C with primary antibodies (Fat CD36 rabbit monoclonal antibody (ab 133625, Abcam, Great Britain) and FABP4/SCL27A4 rabbit polyclonal antibody (ab 666682, Abcam, Great Britain) at 1:1,000. Chemiluminescent detection of proteins was performed following incubation of membranes with horseradish peroxidase-conjugated donkey anti-rabbit IgG antibody (sc 2313; Santa Cruz Biotechnology, Heidelberg, Germany) at 1:10,000. Blots were then exposed to X-ray film (Hyperfilm ECL, Amersham Pharmacia Biotech) and protein expression determined by the ratio of sample band intensity to that of the internal standard (mix of all control group specimens) by densitometry using a GS 800 densitometer controlled by Quantify One 4.6.1 software (Bio-Rad, Marne-La-Coquette, France).

### RNA Isolation, cDNA Synthesis, and Real-Time qPCR and Quantification

A sample of 25 mg taken from the mid-belly of the muscle was disrupted in 50 mg.ml^−1^ Qiazol reagent (Qiagen, Courtaboeuf, France) with a Mixer Mill MM300 (Retsch, Haan, Germany). Total RNA was isolated using an RNAeasy Lipid Tissue Mini kit (Qiagen, Courtaboeuf, France) with an additional DNase step using a Qiacube system (Qiagen, Courtaboeuf, France). Total RNA concentration and purity were assessed by measuring the optical density with a Nanodrop 1000 Spectrophotometer (ThermoFisher Scientific, Wilmington, DE). Reverse transcription was carried out in a 20-μl reaction volume from 800 ng total RNA using the Reverse Transcriptase Core Kit (Eurogentec, Seraing, Belgium), with 50 μM oligo (dT) primers and RNase inhibitor (4 UI). Primer design and optimization in terms of dimerization, self-priming, and melting temperature, were carried out using MacVector software (Accelrys, San Diego, CA).

Primers used in this study (Table 1) were designed from sequences in the flanking introns and then assessed for specificity using the Blast algorithm (https://blast.ncbi.nlm.nih.gov). qPCR was carried out using a LightCycler Fast Start DNA Master SYBR Green kit (Roche Applied Science, Mannheim, Germany). Relative quantification was performed using the comparative threshold method, normalized by geometric averaging against the multiple housekeeping genes CycA, hypoxanthine-guanine phosphoribosyl transferase, and acidic ribosomal phosphoprotein after their stability was validated using Genorm software (Vandesompele et al., 2002).

**Table 1:** Oligonucleotide primers used for real-time PCR amplification.

**Gene**	**Primer probe**	**Sequence**	**Amplicon length (bp)**	**Annealing: duration and temperature (^**°**^C)**	**Gene Bank accession number**
PGC1 α	Forward Reverse	5′-ACGCAGGTCGAATGAAACTGAC-3′ 5′-TGGTGGAAGCAGGGTCAAAATC-3′	116	53° 5 s	NM-031347
PPAR δ	Forward Reverse	5′-TCTGCAAGATCCAGAAGAAGAACC-3′ 5′-GCATCCTTCCAAAGCGGATAG-3′	105	55° 5 s	NM-013141
ARBP	Forward Reverse	5′-CCTGCACACTCGCTTCCTAGAG-3′ 5′-CAACAGTCGGGTAGCCAATCTG-3′	74	55° 3 s	NM-022402
CycA	Forward Reverse	5′-AGCATGTGGTCTTTGGGAAGGTG-3′ 5′-CTTCTTGCTGGTCTTGCCATTCC-3′	92	58° 5 s	M-19533
HPRT	Forward Reverse	5′-CTCATGGACTGATTATGGACAGGAC-3′ 5′-GCAGGTCAGCAAAGAACTTATAGCC-3′	123	58° 5 s	S-79292

### Statistical Analysis

Data are presented as the means ± SEM. Two-way analysis of variance (ANOVA) was used to assess differences between the four experimental groups and identify training and/or hypoxia effects. When appropriate, differences between groups were assessed using a Newman-Keuls *post-hoc* test. Differences were considered significant if *P* < 0.05.

## Results

### Anatomical Data and Hematocrit

As expected in this model, there was no difference in body weight between groups after 5 weeks of training and exposure to hypoxia. Endurance training and hypoxia exposure *per se* tended toward decreasing fat mass (-30%, *P* = 0.09 and −20%, *P* = 0.32, Table 2), whereas, unexpectedly, the relative weight of the *plantaris* muscle to body weight slightly but significantly increased following hypoxia (+6%, *P* = 0.017, Table 2).

**Table 2:** Anatomical data parameters and hematocrit at the end of conditioning.

	**LL**	**LH**	**LLTL**	**LHTH**
Body weight (BW) (g)	226 ± 7	219 ± 5	223 ± 5	222 ± 5
Retroperitoneal fat mass^a^ (g)	1.04 ± 0.18	0.80 ± 0.13	0.70 ± 0.11	0.67 ± 0.08
Plantaris weight^a^ (g)*	88.8 ± 1.6	95.0 ± 2.7	88.4 ± 2.8	93.4 ± 1.6
Left ventricle mass^a^ (g)	193.1 ± 4.3	189.5 ± 3.1	190.1 ± 6.8	188.2 ± 8.8
Right ventricle mass^a^ (g)	55.8 ± 1.9	52.8 ± 2.5	51.7 ± 2.6	62.2 ± 2.1*^$^
Hematocrit (%)	37.7 ± 1.2	46.5 ± 1.1***	39.3 ± 0.4	46.4 ± 0.6***

*Data are expressed as the mean ± SEM. LL, normoxic and sedentary group; LH, hypoxic and sedentary group; LLTL, normoxic and trained group; LHTH, hypoxic and trained group*.

a *Expressed in grams per 100 g body weight*.

Statistically different from the normoxic group with the same level of activity, *P < 0.05 and ***P < 0.001.

*atistically different from the sedentary group in the same environment, ^$^P < 0.05*.

TL had no effect on heart mass, either that of the right or left ventricular. LH did not induce right ventricular hypertrophy in females. Interestingly, right ventricular mass increased exclusively in the LHTH group relative to that of the LH and LLTL groups (+18%, *P* = 0.021 and 20%, *P* = 0.017, respectively, Table 2).

As expected, the hematocrit increased from 38 to 46% (*P* < 0.001, Table 2) with hypoxia in LH and LHTH.

### Aerobic Performance and Plasma Biochemical Parameters After Running

**Figure 1:**
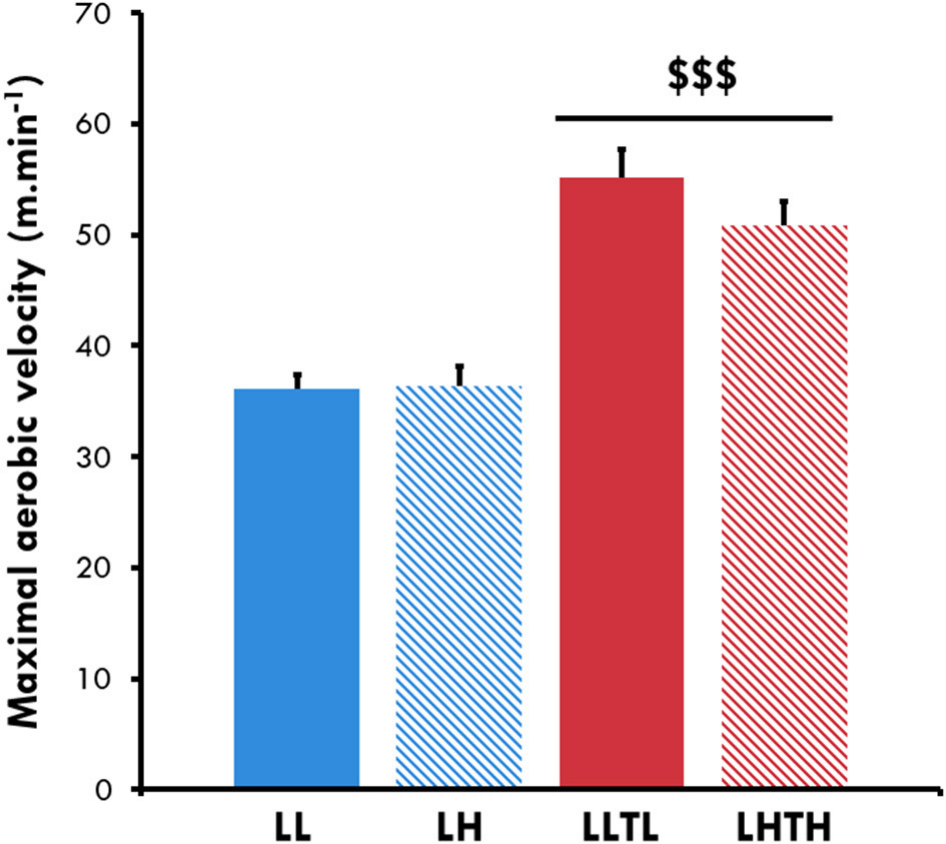
Maximal aerobic velocity at the end of conditioning. ^$$$^Different from the sedentary group in the same environment, *P* < 0.001.

MAV was perfectly comparable between the sedentary groups: 36.1 ± 1.3 m.min^−1^ for LL and 36.3 ± 1.8 m.min^−1^ for LH. Five weeks of training increased the MAV for both the LLTL and LHTH rats (+52 and +39%, respectively, *P* < 0.001, Figure 1), with no significant difference between normoxia and hypoxia. Hypoxia *per se* had no effect on MAV.

**Figure 2:**
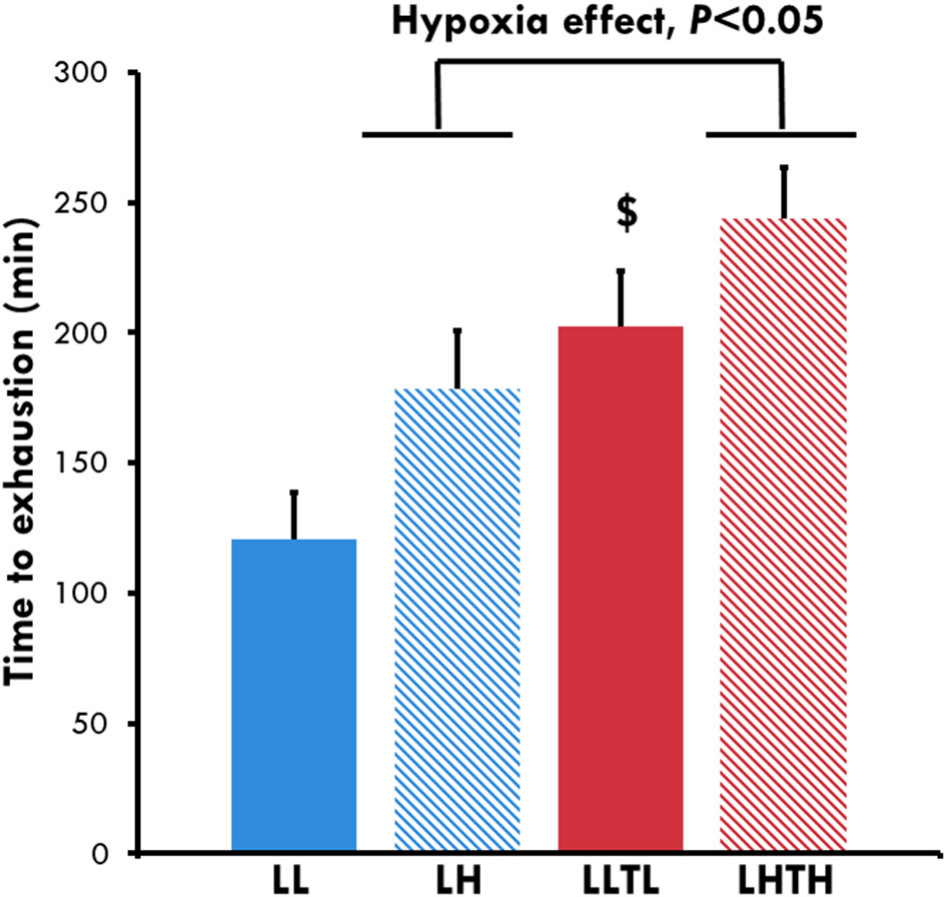
Time to exhaustion at the end of conditioning. ^$^Different from the sedentary group in the same environment, *P* < 0.05.

Hypoxia and training improved the time to exhaustion at 65% of MAV independently of each other (*P* = 0.015 and *P* = 0.009, respectively, Figure 2). The time to exhaustion was 121 ± 21 min for the LL group vs. 178 ± 22 min for the LH for a similar running speed (*P* = 0.054). The time to exhaustion in the LH group was not significantly different from that of the LLTL group (202 ± 21 min), even though the running speed was higher in the trained group. Comparison of the distance run by the animals (Table 3) showed that hypoxia and training have a major effect on performance, with no interaction (*P* = 0.025 and *P* < 0.0001, respectively). The LH group ran 1.5 km more (1.5-fold more) than the LL group (*P* = 0.054), whereas the LHTH group ran 7.98 ± 0.57 km, which is 3.8 km more (thus, 1.8-fold more) than the distance run by the LH group (*P* < 0.001) and 1 km more (+15%, ns) than that run by the LLTL rats. Thus, the effect of hypoxia was additive to training for the distance run at the same relative intensity (65% of MAV), for which the absolute speed was also very similar (36 m.min^−1^ for the LLTL group and 33 m.min^−1^ for the LHTH group).

**Table 3:** Biochemical plasma parameters at the end of the time-to-exhaustion test.

	**LL**	**LH**	**LLTL**	**LHTH**
Distance run (km)*	2.84 ± 0.40	4.38 ± 0.63	6.94 ± 0.51^$$$^	7.98 ± 0.57^$$$^
Free Fatty Acid (μMol.L^−1^)	863 ± 167	1,146 ± 157	703 ± 101	811 ± 90
Glycaemia (mMol.L^−1^)^$^	7.52 ± 0.63	6.50 ± 0.69	7.18 ± 0.34	5.33 ± 0.63
Lactate (mMol.L^−1^)	4.68 ± 0.61	4.31 ± 1.14	5.48 ± 1.11	2.37 ± 0.53

*Data are expressed as the mean ± SEM. LL, normoxic and sedentary group; LH, hypoxic and sedentary group; LLTL, normoxic and trained group; LHTH, hypoxic and trained group*.

Statistically different from the normoxic group with the same level of activity, *P < 0.05.

*Statistically different from the sedentary group in the same environment, ^$^P < 0.05 and ^$$$^P < 0.001*.

At the end of the time-to-exhaustion test, glycaemia was globally lower in the trained than sedentary groups, but this must be considered in light of the duration of the exercise and the distance run, which were longer for the trained (LLTL and LHTH) than sedentary animals (LL and LH) (Table 3). Free fatty-acid levels were not altered in any group, whereas lactate levels in the LHTH group trended being lower than those in the LLTL group (*P* = 0.075).

### Oxidative Capacities in the *Plantaris* Muscle

We evaluated whether the increased endurance capacity induced by the combination of training and hypoxia was associated with mitochondrial adaptations by measuring mitochondrial respiration in permeabilized fibers from the *plantaris* muscle. As expected, endurance training induced an increase in the maximal oxidative rate (Vmax) under normoxia (+22%, *P* = 0.028, Table 4) but not in hypoxia, resulting in a 23% lower Vmax (*P* = 0.009) in the LHTH than in the LLTL group. We measured the enzymatic activity of CS to evaluate whether this effect resulted from changes in the mitochondrial mass. We showed an increase in CS activity in both the LLTL and LHTH groups relative to that in their respective sedentary groups (*P* < 0.001). Hypoxia limited the improvement in CS induced by training (+40% for LLTL vs. +25% for LHTH relative to their respective sedentary groups; interaction *P* = 0.0016) and resulting in an 30% lower activity in the LHTH than in LLTL group (*P* < 0.001). Hypoxia exposure *per se* significantly decreased CS activity in the sedentary animals (−22%, *P* < 0.001). After 5 weeks of training, mRNA levels for proliferator-activated receptor-gamma coactivator 1 alpha (PGC-1α) were not yet elevated regarless of environment (**Figure 4**).

**Table 4:** Maximal muscle oxidative capacity, citrate synthase and 3-hydroxyl-acyl coenzyme A dehydrogenase activities in the *plantaris* muscle.

	**LL**	**LH**	**LLTL**	**LHTH**
Vmax^a^ (μmole O_2_.min^−1^.g^−1^)	12.3 ± 0.7	13.5 ± 0.7	15.0 ± 0.8^$^	11.6 ± 0.7**
CS^b^ (UI.g^−1^)	44.2 ± 0.9	34.3 ± 1.3***	61.6 ± 1.2^$$$^	43.4 ± 1.4^$$$^***
3-HAD^b^ (UI.g^−1^)	3.45 ± 0.19	3.24 ± 0.34	6.34 ± 0.56^$$$^	5.35 ± 0.51^$$$^

*Data are expressed as the mean ± SEM. LL, normoxic and sedentary group; LH, hypoxic and sedentary group; LLTL, normoxic and trained group; LHTH, hypoxic and trained group; Vmax, maximal oxygen consumption in the presence of saturating concentrations of ADP, glutamate, and succinate; CS, citrate synthase; 3-HAD, 3 hydroxy-acyl coenzyme A dehydrogenase*.

a *Vmax values are expressed as μmole O_2_.min^−1^.g^−1^ of dry weight*.

b *Enzymatic activity is expressed in UI.g^−1^ of wet weight*.

Statistically different from the normoxic group for the same level of activity, **P < 0.01 and ***P < 0.001.

*Statistically different from the sedentary group in the same environment, ^$^P < 0.05 and ^$$$^P < 0.001*.

Overall, our results show that the increased endurance induced by the combination of training and hypoxia was not associated with a greater increase in oxidative capacity or mitochondrial mass of the skeletal muscle in the LHTH group.

### Mitochondrial Substrate Utilization and Mitochondrial Efficiency in the *Plantaris* Muscle

**Figure 3:**
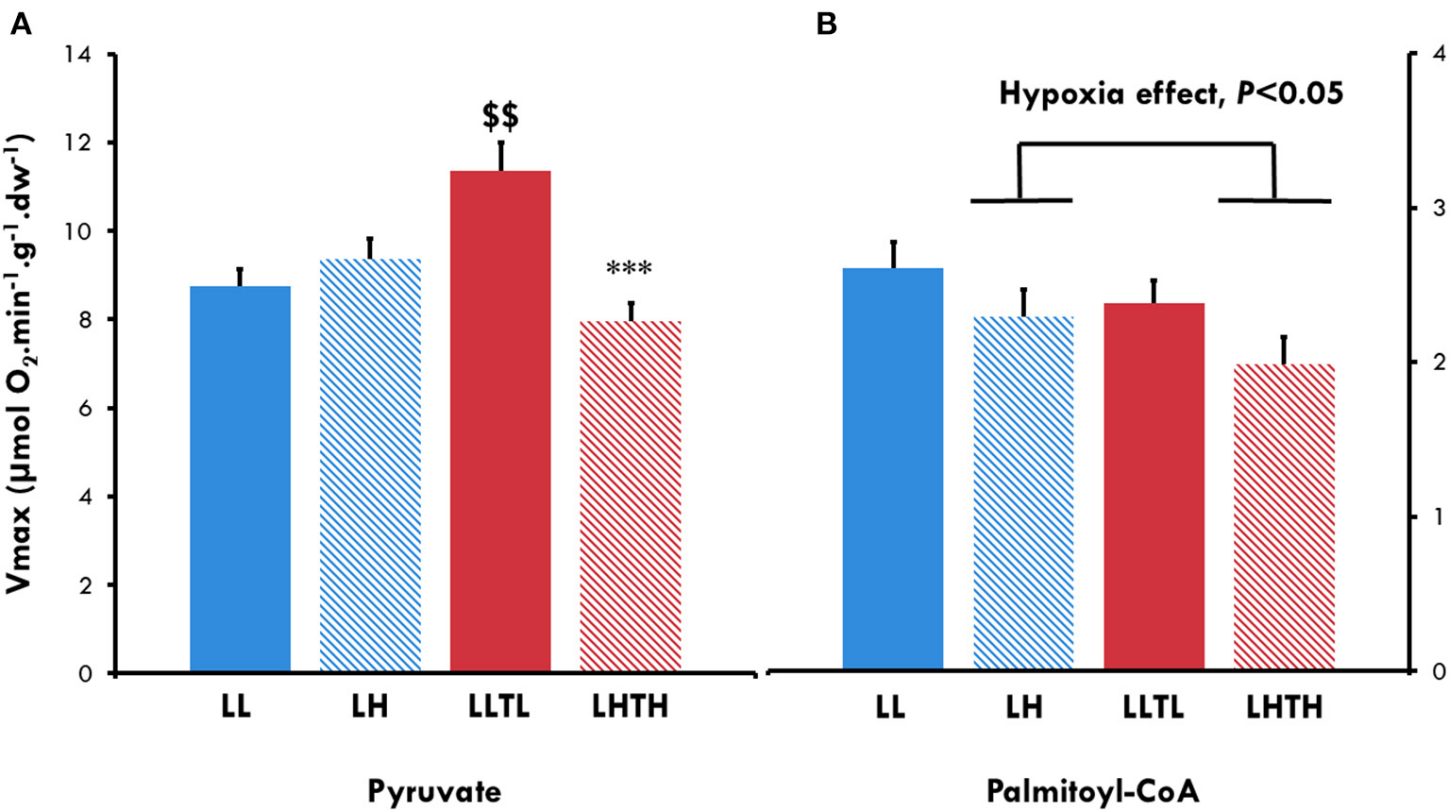
Maximal oxidative capacity for pyruvate **(A)** and Palmitoyl-CoA **(B)** in *plantaris* skinner fibers of female rats at the end of conditioning. ^$$^Different from the sedentary group in the same environment, *P* < 0.01. ***Different from the normoxic group with the same level of activity, *P* < 0.001. Successive addition of 2 mM pyruvate, 4 mM malate, 2 mM ADP, and 20 mM creatine for pyruvate and successive addition of 400 μM Palmitoyl-CoA in the presence of 2 mM ADP, 0.5 mM malate, and 1 mM carnitine for Palmitoyl-CoA.

We evaluated whether the increased endurance induced by the combination of training and hypoxia was associated with a shift in mitochondrial substrate utilization by measuring mitochondrial respiration in the presence of pyruvate and Palmitoyl-CoA, respectively, a glycolytic and lipid substrates. LLTL improved maximal respiration with pyruvate by 31% (*P* = 0.0019). Conversely, LHTH did not increase the V_maxPyr_, which was 40% lower than that of the LLTL group (*P* < 0.001, Figure 3), as already observed for the maximal rate of oxidative capacity with malate-glutamate-succinate. Hypoxia globally decreased the Vmax specifically for Palmitoyl-CoA (V_maxPCoA_), whereas we did not find a training effect (Figure 3).

3-HAD activity was altered only by training, independently of the environment: +83% in the LLTL group and +64% in the LHTH group (*P* < 0.001) relative to sedentary rats (Table 4).

**Figure 4:**
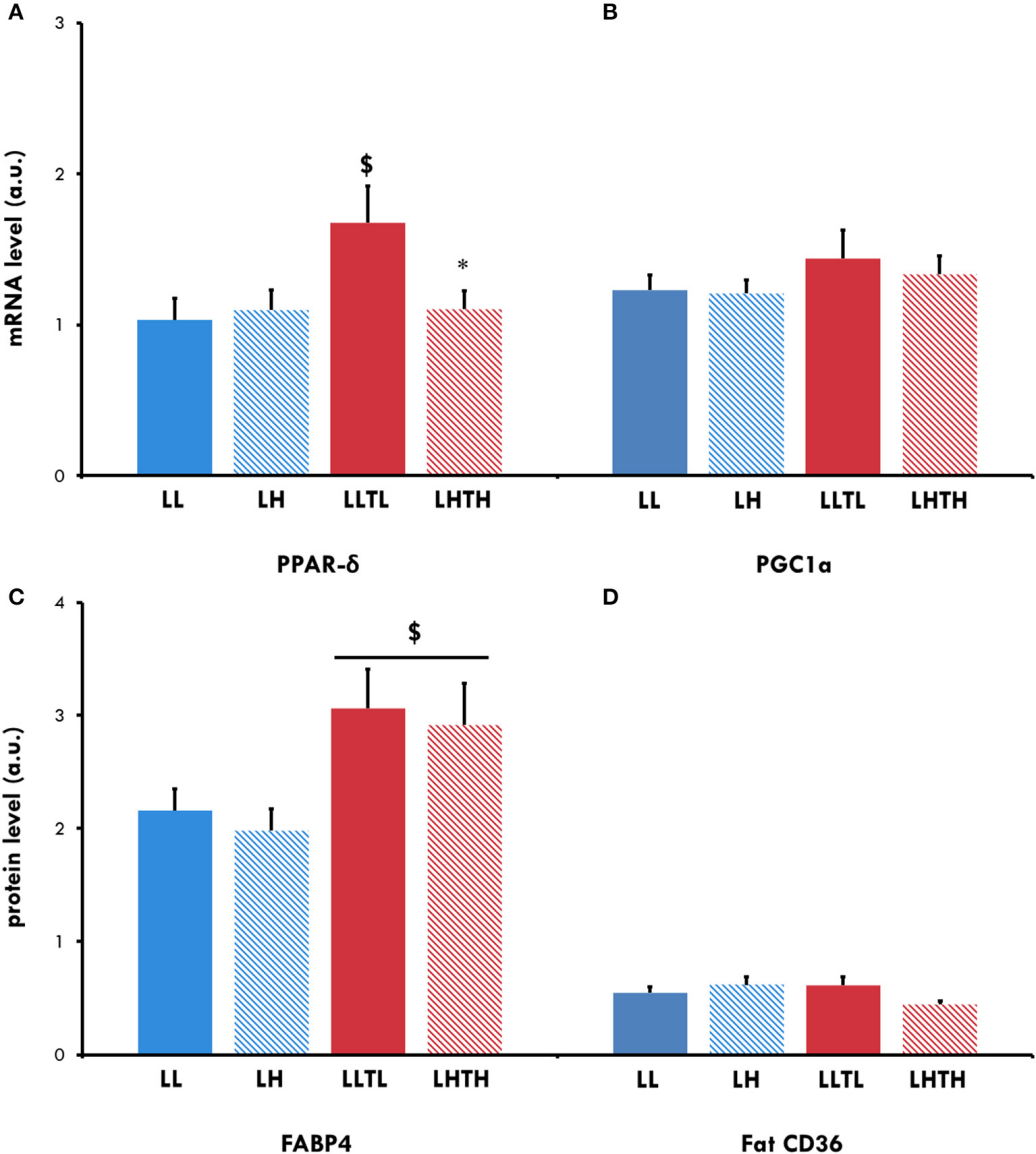
Relative mRNA expression for PPAR-δ **(A)** and PGC-1α **(B)** and protein levels for FABP4 **(C)** and Fat CD36 **(D)** in the *plantaris* muscle of female rats. ^$^Different from the sedentary group in the same environment, *P* < 0.05. *Different from the normoxic group with the same level of activity, *P* < 0.05.

PPAR-δ mRNA expression increased in the LLTL group (*P* = 0.042) but not the LHTH group (Figure 4). Protein expression of Fat CD36, one of its targets and a sarcolemmal transporter of FA, was not altered by training or hypoxia but there was almost an interaction (*P* = 0.056), with a trend for hypoxia to decrease Fat CD36 expression when associated with training. FABP-4 protein, another sarcolemmal FA transporter, globally increased to a similar extent after training (*P* = 0.0042) under hypoxia (LHTH) or normoxia (LLTL) (Figure 4).

We evaluated whether the increased endurance induced by LHTH was associated with improved mitochondrial efficiency (Table 5) by studying the acceptor coupling ratio (ACR) and found it lowered by hypoxia (−27%, *P* < 0.001). The classical increase of Km for ADP induced by training was suppressed by concomitant exposure to hypoxia (interaction training x hypoxia *P* = 0.04). Moreover, as expected, the creatine kinase efficiency (estimated Km ADP without creatine/estimated Km ADP with creatine) was increased in LLTL (*P* = 0.005) but not in LHTH, and was 50% lower in the LHTH compared to the LLTL group (*P* = 0.015).

**Table 5:** Mitochondrial markers of efficiency in the *plantaris* muscle.

	**LL**	**LH**	**LLTL**	**LHTH**
ACR***	3.46 ± 0.46	2.86 ± 0.14	3.70 ± 0.19	2.36 ± 0.06
^a^Km ADP ^interaction hypoxia × *training, *P** < 0.05^	100.3 ± 11.8	122.9 ± 15.3	124.7 ± 5.8	94.8 ± 12.8
^a^Km ADP + creat	23.4 ± 2.5	28.0 ± 5.2	20.4 ± 2.3	23.1 ± 2.8
Ratio Km ADP/Km ADP+ creat	3.56 ± 0.56	3.50 ± 0.84	6.37 ± 0.99^$$^	3.33 ± 0.27*

*Data are expressed as the mean ± SEM. LL, normoxic and sedentary group; LH, hypoxic and sedentary group; LLTL, normoxic and trained group; LHTH, hypoxic and trained group*.

a *Km values are given as the concentration of ADP expressed in μM*.

Statistically different from the normoxic group with the same level of activity, * P < 0.05 and ***P < 0.001.

*Statistically different from the sedentary group in the same environment, ^$$^*P* < 0.01*.

## Discussion

### Major Findings

Five weeks of endurance training increased MAV and time to exhaustion of female rats at a relative, moderate intensity. Living and training under moderate hypoxia (LHTH) showed no additional benefit on MAV but extended the time to exhaustion and thus the distance run. There was an additive effect of hypoxia with training for sustained aerobic performance at a submaximal level (65% MAV). In the context of this moderate level of intensity, we had hypothesized that muscle metabolic adaptations, particularly fatty-acid utilization, would have been improved by LHTH. Our current results exclude the participation of mitochondrial alterations and glycogen sparing by facilitation of fatty-acid utilization in the observed improvement in endurance and resistance to fatigue.

The main novelty of our results is the improvement of endurance in LHTH relative to LLTL, despite the fact that all quantitative and qualitative training-induced mitochondrial adaptations were limited by hypoxia. We confirmed that hypoxia impedes the maximal oxidation of Palmitoyl-CoA, does not improve β-oxidation or 3-HAD activity and accentuates glucose dependence, as already described (Brooks et al., 1991; Roels et al., 2007). Hypoxia *per se* also decreased coupling between oxidation and phosphorylation. Moreover, hypoxia impaired normal training-induced mitochondrial responses, such as increased mitochondrial mass, maximal oxidative capacity (CS and Vmax) and the subsequent increase of maximal oxidation of pyruvate. Other qualitative mitochondria alterations normally observed with endurance training were also diminished, such as increased efficiency of creatine kinase and PPAR-δ mRNA expression.

These elements suggest that mitochondrial function and, specifically, fatty-acid utilization, do not contribute to the better aerobic performance in long-distance running observed after LHTH.

### Strengths and Limitations

Here, we observed a large effect of our training program on aerobic performance, with classical training-induced muscle adaptations, which is a good starting point to evaluate the additive or negative effects of such a program with hypoxia and discuss ergogenic effects and their origin. Human studies, comparing LHTH and LLTL, are often conducted with athletes and improved performance has not always been observed and classical muscle adaptations induced by training rarely present in normoxia, limiting the interpretation of the combined effect of hypoxia and training (Robach et al., 2014).

Studies conducted on the metabolic alterations induced by LHTH have been primarily conducted using male rats. However, we voluntarily designed our study using females to limit the deleterious effect of hypoxia on the energy balance (Wood and Stabenau, 1998), which is often a bias in severe and prolonged exposure of males to hypoxia. In the model used here, we observed no difference in body weight, often induced by hypoxia or training in male rats (Foright et al., 2020), limiting confounding metabolic factors due to differences in food intake. We recognize that rats exhibit sexual dimorphism in basal mitochondrial mass, substrate utilization and response to exercise (Lundsgaard and Kiens, 2014; Ventura-Clapier et al., 2017), which could also interfere with the magnitude of the effect of hypoxia independently of the effect on the energy balance. Thus, it cannot be excluded that the impediment of metabolic adaptations would have been different in males. Nevertheless, in light of the results from two studies conducted on high altitude-acclimatized women (Braun et al., 2000) and female rats (McClelland et al., 1998), we assume that the female model would show limited consequences of hypoxia on the impediment of fatty-acid metabolism observed in men (Brooks et al., 1991; Roberts et al., 1996) which strenghtens our results.

We chose to adapt the running speed to maintain a similar relative intensity during the TH sessions relative to TL. Thus, the absolute intensity was lower during the TH, which could constitute a bias independent of the hypoxic condition. It is noteworthy that studies applying a “same relative intensity” approach have often failed to show additional effects on the oxidative capacity of muscle and performance, whereas differences could be found when TH and TL were performed at the same absolute intensity (Vogt and Hoppeler, 2010). The major hypothesis to explain such a lack of improvement observed with TH is related to the decreased intensity because of the lower O_2_ flow, corresponding to a type of relative detraining (Levine, 2002). Indeed, it has been clearly shown that exercise intensity *per se* highly correlates with muscle mitochondrial content (Jacobs et al., 2013b) and thus lower intensity could decrease the magnitude of mitochondrial biogenesis. It cannot be excluded that higher intensity would have partially compensated for the smaller adaptation of mitochondrial mass observed here. This observation is at the origin of a new TH strategy consisting of repeated sprints in hypoxia, in which short “all-out” efforts can be completed at the same velocity as in normoxia. Such an approach appears to induce additional molecular adaptations of skeletal muscle relative to a similar level of exercise in normoxia (Faiss et al., 2013). Nevertheless, the same reasoning cannot be applied to fatty-acid utilization and β-oxidation, which are more highly stimulated by lower intensity (Achten and Jeukendrup, 2003) and should have been facilitated by the reduction of intensity during TH. Finally, as there was an improvement in the time to exhaustion with LHTH, despite the lower absolute intensity of our hypoxic-training protocol, it is still relevant to discuss the contribution of metabolic adaptations to the observed increase in endurance.

Due to material constraints, we were unable to perform training in hypobaric hypoxia while animals were living under hypobaric conditions. We admit that this is not representative of the reality of a natural altitude training camp. If differences in physiological alterations have already been described between normobaric and hypobaric acute hypoxia exposure (Savourey et al., 2003), the same changes in performance after LHTL in normobaric vs. hypobaric hypoxia have been published (Saugy et al., 2016).

Our primary aim was to describe mitochondrial alterations with LHTH and, thus, we did not focus on the erythropoietic effect. We only measured the hematocrit to internally validate our intervention but did not properly measure changes in Hbmass, which limits the consideration of this hypothesis.

Finally, as our intervention was long term and we were unable to remove tissue under hypoxic conditions, we did not measure HIF-1α protein levels, which could have been informative. However, we did not find any elevation in mRNA levels of HIF-1 target genes, such as VEGF (data not shown).

### Less Improvement of Oxidative Capacity and Mitochondrial Mass After LHTH Than After LLTL

Exposure to hypoxia, even at a moderate level, limited the increase of mitochondrial mass and maximal oxidative capacities induced by endurance training. Here, LH *per se* decreased CS activity, whereas previous studies did not find such alterations with hypoxia in male rats (Bigard et al., 1991; Daneshrad et al., 2000; De Palma et al., 2007; Galbes et al., 2008; Jacobs et al., 2012b; Malgoyre et al., 2017). Classically, alterations of mitochondrial mass have been reported for higher levels of hypoxia and typically described for mountaineers during Himalayan expeditions of several weeks (Jacobs et al., 2012b; Levett et al., 2012). Nevertheless, this decrease in CS activity was not associated with alteration of the maximal respiration rate for glutamate-succinate, suggesting that mitochondrial function is preserved in LH and that CS is not a limiting factor of respiration in sedentary female muscle. Surprisingly, our results contradict those of a Jacobs et al. (2012b) in human males, in which they observed no alteration of *vastus lateralis* mitochondrial mass after 1 month at 3,454 m, whereas respiratory capacity in this muscle was reduced.

The most evident sign of the blunting of mitochondrial biogenesis by hypoxia was the limited increase in CS induced by endurance training. Here, the observed lower increase in CS is completely consistent with the lower Vmax in LHTH relative to LLTL (−29%, *P* < 0.001). As CS is now considered to be a good biomarker of mitochondrial content (Larsen et al., 2012), we assume that the lower oxidative capacities observed in LHTH vs. LLTL are essentially related to a smaller increase of mitochondrial mass, corresponding to less intense mitochondrial biogenesis. Four weeks of simulated LH, equivalent to 3,000 m, did not modify the maximal capacity of oxidative phosphorylation in TL athletes (Robach et al., 2012). Two companion papers on elite male team-sport players reported, after 14 days of LHTL, a large increase in succinate dehydrogenase (SDH) levels, likely higher than in LLTL (van der Zwaard et al., 2018), whereas the training-induced increase of CS was abolished by LHTL (Brocherie et al., 2018). Because of the dissociation between the responses of SDH and CS in these two studies, it is difficult to draw conclusions concerning the effect of LHTL in this human study. Furthermore, these two papers focused on a third condition, adding repeated sprints in hypoxia to LHTL, which restored the CS and further increased SDH responses to TL.

Among the only two studies that investigated LHTH in rat models, neither reported such a limitation with hypoxia and, instead, an even greater increase in CS levels in the *plantaris* was observed after LHTH (Bigard et al., 1991; Galbes et al., 2008). A gender effect cannot be excluded, as male rats were used for these studies, whereas we used females. Although we cannot exclude that the mitochondria of female rats are more severely altered by hypoxia than those of male rats, the most obvious explanation is that the negative energy balance in male rats (known to facilitate biogenesis, including activation of AMPK and Sirt-1 signaling), supported by their lower body weight at the end of the altitude intervention, could have partially compensated for the negative effect of hypoxia on metabolic adaptation induced by training.

At the cellular level, PGC-1α mRNA levels were not yet elevated after 5 weeks of training, which is not surprising, because the increase of oxidative capacity resulting from mitochondrial biogenesis had already occurred. We did not observe any differences between LHTH and LLTL, whereas a number of human studies on LLTH found a larger increase in mitochondrial biogenesis transcription factors or the mRNA of oxidative and respiratory enzymes (Vogt et al., 2001; Zoll et al., 2006) after TH than TL. Our results are consistent with other study in humans, where no alteration of several mitochondrial biogenesis transcription factors levels was found after 2 weeks of LHTL (Brocherie et al., 2018).

Our results are entirely consistent with the role of HIF-1 described in skeletal muscle and the assumption that high levels of HIF-1α may have a strong negative effect on mitochondrial adaptation after endurance training. The observed downregulation of HIF-1 found after endurance training (Lindholm and Rundqvist, 2016) and basal high level of oxidative capacity in skeletal muscle of HIF-1 null mice (Mason et al., 2007) are in accordance with our results. Recently, Favier et al. (2016) found that pharmacological inhibition of hydroxylase, responsible for HIF-1 stabilization, decreased the maximal oxidative rate in mice gastrocnemius muscle.

Nevertheless, we cannot exclude that training at the same relative intensity between TH and TL in our study, which represents an absolute intensity that is 20% lower under TH than TL, *per se* limited the increase in mitochondrial mass (Lundby and Jacobs, 2016), independently of any effect of hypoxia.

### Pyruvate and Palmitoyl-CoA Oxidation Do Not Increase After LHTH

#### Lower Increase in Pyruvate Oxidation After LHTH

The alteration of pyruvate oxidation in the LHTH group relative to that in the LLTL group correlates well with the smaller increase in oxidative capacities observed in the LHTH group and does not support the specific alteration of pyruvate oxidation. This appears to be the consequence of a smaller mitochondrial mass in this group, as suggested by the lack of impediment of relative pyruvate oxidation after LHTH.

#### Decrease in Long-Chain Fatty Acid Oxidation Under Hypoxia

Despite the observed decrease in CS activity, we found no significant degradation of 3-HAD activity (the limiting β-oxidation enzyme) in LH and the response to endurance training was well-preserved relative to that of the citric-acid enzymes (LHTH). These results are consistent with those of Bigard et al. (1991), who showed an increase in 3-HAD activity in the rat *plantaris* after LHTH.

The already reported lack of alteration of oxidation for octanoyl carnitine in humans (Jacobs et al., 2012b) or palmitoyl carnitine in rat slow-twitch muscle (Malgoyre et al., 2017) after prolonged exposure to hypoxia also supports the preservation of β-oxidation.

Nevertheless, we found that hypoxia specifically decreases V_maxPCoA_, as already reported in fast/glycolytic (Malgoyre et al., 2017) or mixed muscle (Galbes et al., 2008) after exposure to hypoxia, as well as in humans after LLTH (Roels et al., 2007). Thus, the *plantaris*, a glyco-oxidative muscle, shows a response to hypoxia that is more similar to that of fast/glycolytic. These results are supported by lower CPT-1 protein levels and activity found in rodent skeletal muscle (Kennedy et al., 2001; Galbes et al., 2008; Morash et al., 2013) after chronic exposure to hypoxia.

This is consistent with the absence of an increase in PPAR-δ mRNA levels in LHTH, as CPT-1 is one of the targets of this nuclear factor. Fat CD36 barely showed a tendency to decrease in LHTH and the levels of FABP-4 protein, another target of this transcription factor, globally increased after training (*P* < 0.005), but similarly in LHTH and LLTL. Thus, sarcolemmal transport of fatty acids may not be limited by hypoxia or to a lesser extent, contrary to mitochondrial transport.

Neither training nor hypoxia had a significant effect on FFA measured at the end of the time-to-exhaustion test. Nevertheless, the time to exhaustion for the LH group was quite similar as that of the LLTL group, whereas FFA levels were almost twice as high (*P* = 0.29). Such an increase is consistent with lower fatty-acid utilization (Roberts et al., 1996) and increased dependence on blood glucose, described in humans after altitude acclimatization (Brooks et al., 1991). The decrease in glycaemia levels with training can probably be explained by the longer duration and distance run by these groups. The tendency of lactate to decrease in LHTH vs. LLTL (*P* = 0.08) suggests the activation of neo-gluconeogenesis and the use of lactate as a substrate, probably to compensate for the lower fatty-acid utilization.

### OXPHOS Efficiency, Uncoupling, and Mitochondrial Creatine Kinase

This was a very secondary aim of this study and only the ACR for pyruvate was calculated and found to decrease in response to hypoxia. The literature is highly divergent on the subject of mitochondrial efficiency after exposure to hypoxia. All possible results have been reported: no alteration (Ponsot et al., 2006; Bakkman et al., 2007; Roels et al., 2007; Jacobs et al., 2013a), improvement (Jacobs and Lundby, 2013), or decrease of coupling for fatty acids (Robach et al., 2014). The group of Gnaiger found that FCCP (an uncoupling drug) did not increase the respiration rate after TH vs. TL in humans, suggesting partial uncoupling of respiration in the TH group (Pesta et al., 2011) consistent with our data and the reported increase of UC3 expression (Lu and Sack, 2008; Levett et al., 2012). Recently, substantial uncoupling (ACR divided by two) was found in skeletal muscle after pharmacological stabilization of HIF-1 in mice (Favier et al., 2016) but this does not represent a physiological context.

Alteration of the metabolic phenotype has also been described with a shift to a more oxidative profile after TH in Olympic athletes and characterized by an increase of Km for ADP (Ponsot et al., 2006). Here, we obtained a different result, as induction of a more oxidative profile by training (elevation of Km for ADP and mitochondrial creatine kinase efficiency), was limited with LHTH.

Finally, given these results, it is very unlikely that fatty-acid utilization improves endurance after LHTH, and nothing suggests better OXPHOS efficiency after LHTH. Nevertheless, despite such a decrease in mitochondrial mass and function when implementing LHTH, maximal aerobic performance was not altered, and endurance was even improved relative to LLTL.

### Hypothesis for the Improvement of Endurance After LHTH

Despite poor mitochondrial adaptation, the time to exhaustion at 65% of MAV was significantly increased by hypoxia, independently of training but with an additive effect. Hypotheses other than mitochondrial alterations can be put forward to explain such an improvement in the time to exhaustion following hypoxia, as suggested by certain morphological measurements. Other more putative mechanisms, not addressed in this study, are also possible, such as increased erythropoiesis, angiogenesis, or left and right cardiac function.

The increase in endurance cannot be related to the lower body weight for the hypoxic groups often reported in other studies (Bigard et al., 1991; Galbes et al., 2008). However, the mass of the *plantaris* was significantly higher in the hypoxic groups (*P* < 0.005) and could indirectly contribute to the improved performance in the LHTH group due to an increase in active muscle mass.

Similarly, we found hypertrophy of the right ventricle (HRV) in the LHTH group. If HRV is a classical consequence of chronic severe hypoxia (Koulmann et al., 2006), more moderate hypertrophy could be determinant for long-distance performance and could limit the cardiac fatigue and acute dysfunction of the right ventricle sometimes described at the end of ultra-endurance exercise (Oxborough et al., 2011; Lord et al., 2015). The HRV specifically found in our LHTH group could contribute to the ability of rodents to perform continuous exercise for more than 4-h, and it might have been informative to assess cardiac function at the end of the time to exhaustion, in particular, as female sex hormones limit excessive pulmonary hypertension during chronic hypoxic exposure (Ou et al., 1994).

The correlation of Hbmass with time-trial performance in highly-trained endurance athletes is well-established (Jacobs et al., 2011). We observed a large increase in hematocrit after exposure to hypoxia. Hematocrit alone cannot be considered to be a relevant marker of erythropoiesis. However, as blood was withdrawn 48-h after the time-to-exhaustion trial, we consider that dehydration did not play a major role in this situation and that the elevation of hematocrit largely represents an increase in hemoglobin levels. Even when properly measured, the increase in Hbmass in elite athletes after LH has been vigorously debated (Robach et al., 2012; Millet et al., 2019). It has been suggested by some that the intensity of erythropoiesis observed with LH may depend on basal Hbmass (Robach et al., 2012), although others argue against this hypothesis (Hauser et al., 2018). A recent study focusing on this issue compared Hbmass responses following LHTH in elite athletes. They showed that female distance runners had greater increases in Hbmass than male distance runners during altitude training (Heikura et al., 2018). Moreover, females not only have a lower Hbmass than males but also lower iron stores. As hepcidin that limits intestinal absorption of iron is a sensitive oxygen-responsive gene inhibited by HIF-1 (Hintze and McClung, 2011), hypoxia could also be particularly favorable for increasing iron stores in females, which is required for efficient erythropoiesis.

A very interesting study was performed that addressed the same issue that we did. Ten athletes were assigned to LHTL: half showed a slight increase in Hbmass that did not always correlate with a small increase in VO_2max_, but all of which were abolished by isovolumic hemodilution (Robach et al., 2012). In addition, none of the athletes showed an increase in CS activity or pyruvate oxidation. Although the authors of this study were focused on demonstrating the absence of a relationship between increased VO_2max_ and increased Hbmass, our interpretation of their results is different and similar to that of our findings. Our results are also consistent with those of Favier et al. (2016) and suggest that the major effect observed in a pharmacological hypoxia mimetic model in mice improved endurance concerns Hbmass, whereas uncoupling occurred in skeletal muscle.

Independently of mitochondria adaptations, an increase in muscle capillarization has been shown to contribute to better oxygen diffusion and improved endurance following LLTH in human (Schmutz et al., 2010; Desplanches et al., 2014). Although we did not evaluate muscle capillarization, we found no differences in the expression of indirect biomarkers of angiogenesis (VEGF and PECAM) in response to hypoxia or training (data not shown) and this hypothesis is still supposed.

## Conclusion

Overall, the results presented here show that mitochondrial adaptations are not involved in the improvement of submaximal aerobic performance observed when combining training and hypoxic exposure. Indeed, mitochondrial adaptation to training was blunted by hypoxia, suggesting that the benefits of altitude camps rely essentially on the transitory elevation of hematocrit and should be planned a few weeks before competition and not several months. These results highlight the need to find the optimal “hypoxic dose” that allows an increase in hemoglobin mass while preventing the impediment of mitochondrial adaptation. Moreover, sex differences should be considered to assess the benefits expected of such a strategy to optimize aerobic performance through interactions between training and environment. Finally, such a lack of improvement of mitochondrial function after LHTH does not exclude the benefits suggested by other types of altitude training, in particular, repeated sprints in hypoxia.

## Data Availability Statement

The raw data supporting the conclusions of this article will be made available by the authors, without undue reservation.

## Ethics Statement

The animal study was reviewed and approved by Comité d'éthique animal du Service de Santé des armées.

## Author Contributions

AMa, HS, and XB designed the study. AMa, AP, BS, RC, AMe, and HS performed the study and collected the data. AMa, AP, and HS analyzed the data. AMa and AP wrote the manuscript. HS, AP, NK, and XB edited the manuscript. All authors contributed to the article and approved the submitted version.

## Conflict of Interest

The authors declare that the research was conducted in the absence of any commercial or financial relationships that could be construed as a potential conflict of interest.

## Abbreviations

BSA: bovine serum albumin
CS: citrate synthase
FFA: free fatty acid
HAD: hydroxyl-acyl-dehydrogenase
Hbmass: hemoglobin mass
HIF: hypoxia-inducible factor
HRV: hypertrophy of the right ventricle
LH: living high
LHTH: living high training high
LL: living low
LLTL: living low training low
MAV: maximal aerobic velocity
PGC-1α: proliferator-activated receptor-gamma coactivator 1 alpha
VEGF: vascular endothelial growth factor
SDH: succinate dehydrogenase.
